# Development of Primer Panels for Whole-Genome Amplification and Sequencing of Human Seasonal Coronaviruses: hCoV-OC43, hCoV-HKU1, hCoV-229E, and hCoV-NL63

**DOI:** 10.3390/v17010013

**Published:** 2024-12-25

**Authors:** Tamila Musaeva, Artem Fadeev, Maria Pisareva, Veronika Eder, Andrey Ksenafontov, Margarita Korzhanova, Valery Tsvetkov, Alexander Perederiy, Irina Kiseleva, Daria Danilenko, Dmitry Lioznov, Andrey Komissarov

**Affiliations:** 1 Smorodintsev Research Institute of Influenza, 197376 Saint Petersburg, Russia; tamila.musaeva@influenza.spb.ru (T.M.);; 2 Institute of Experimental Medicine, 197022 Saint Petersburg, Russia; 3 Department of Infectious Diseases and Epidemiology, First Pavlov State Medical University, 197022 Saint Petersburg, Russia

**Keywords:** viruses, human seasonal coronaviruses, acute respiratory infections, hCoV-NL63, hCoV-229E, hCoV-HKU1, hCoV-OC43, molecular virology

## Abstract

Human seasonal coronaviruses (hCoVs) are a group of viruses that affect the upper respiratory tract. While seasonal patterns and the annual variability of predominant hCoV species are well-documented, their genetic and species diversity in St. Petersburg and across Russia remains largely unexplored. In this study, we developed a two-pool, long-amplicon (900–1100 bp) PCR primer panel for the whole-genome sequencing of four seasonal hCoV species. The panel was validated using nasopharyngeal swab samples collected within the Global Influenza Hospital Surveillance Network (GIHSN) project. Over a period of six epidemiological seasons from 2017 to 2023, we retrospectively analyzed 14,704 nasopharyngeal swabs collected from patients hospitalized in St. Petersburg clinics. Of these samples, 5010 (34.07%) tested positive for respiratory viruses, with 424 (2.88% of all samples) identified as seasonal human coronaviruses. The assessment of species diversity showed that predominant hCoV species alternate between seasons. Whole-genome sequences for 85 seasonal human coronaviruses (hCoVs) with >70% genome coverage were obtained, including 23 hCoV-OC43, 6 hCoV-HKU1, 39 hCoV-229E, and 17 hCoV-NL63. These represent the first near-complete genomes of seasonal hCoVs from the Russian Federation, addressing a significant gap in the genomic epidemiology of these viruses. A detailed phylogenetic analysis of the sequenced genomes was conducted, highlighting the emergence of hCoV-229E subclades 7b.1 and 7b.2, which carry numerous substitutions in the Spike protein. Additionally, we sequenced a historical hCoV-229E isolate collected in the USSR in 1979, the oldest sequenced 229E virus from Eurasia, and demonstrated that it belongs to Genotype 2. The newly developed PCR-based sequencing protocol for seasonal hCoVs is straightforward and well-suited for genomic surveillance, providing a valuable tool to enhance our understanding of the genetic diversity of human seasonal coronaviruses.

## 1. Introduction

The first human coronavirus, B814, was identified in 1965 by a team of British virologists led by David Tyrrell [1]. Later, the term “coronavirus” was introduced, reflecting its distinctive morphology under an electron microscope [2]. The ICTV classification now places human coronaviruses under the order Nidovirales, suborder Cornidovirineae, family Coronaviridae, and subfamily Orthocoronavirinae. This subfamily includes four genera: Alphacoronaviruses, Betacoronaviruses, Gammacoronaviruses, and Deltacoronaviruses [3].

Currently, four types of human seasonal coronaviruses (hCoV) have been described: hCoV-229E, hCoV-OC43, hCoV-NL63, and hCoV-HKU1. Coronaviruses hCoV-229E and hCoV-OC43 were discovered in the 1960s [4,5]. Two more seasonal coronaviruses—hCoV-NL63 and hCoV-HKU1—were reported in the early 2000s [6,7]. Most recently, three significantly more pathogenic strains appeared, namely SARS-CoV [8], MERS-CoV [9], and SARS-CoV-2 [10]. The frequent emergence of new coronaviruses sparked growing interest in exploring these pathogens, their impact on global health, and the mechanisms driving their variability.

While human coronaviruses were officially identified in 1965, some publications suggest they emerged earlier. Thus, Leen Vijgen and colleagues showed that the most recent common ancestor of hCoV-OC43 and bCoV likely appeared in the 19th century. The authors speculate that the virus was first transmitted from cattle to humans around that time, which is supported by records of increased cattle mortality and slaughter that coincided with a human pandemic [11]. Molecular clock analysis dates the most recent common ancestor of bCoV and hCoV-OC43 to around 1890 [12]. In addition to bCoV, hCoV-OC43 is similar to porcine hemagglutinating encephalomyelitis virus (PHEV) in its entire genome sequence, except for the Spike (S) gene [13]. Higher genetic distance in the S region may be attributed to recombination, which is characteristic of all coronaviruses, particularly PHEV [14].

Phylogenetic analysis suggests an evolutionary origin of hCoV-NL63 and hCoV-229E in bats, while hCoV-OC43 and hCoV-HKU1 viruses likely originated in rodents [15,16,17]. Bat coronavirus ARCoV.2, detected in North American tricolored bats, is phylogenetically close to hCoV-NL63. Similarly, HCoV-229E appears genetically related to another bat CoV detected in Ghana, although camelids have also been suspected as its intermediate host. Unlike other coronaviruses, the hCoV-OC43 Betacoronavirus originated from rodent viruses, with cattle considered the intermediate host [18].

The zoonotic origins of human coronaviruses, their replication mechanisms, and the existence of intermediate hosts indicate that novel coronaviruses with high pathogenic potential in humans may emerge in the future. Although all human coronaviruses share a similar replication cycle, they differ in their additional proteins, incubation periods, and pathogenic potential [19,20,21].

Unfortunately, there is limited literature on the circulation of seasonal human coronaviruses [22,23,24,25,26,27,28,29,30,31,32,33]. While there are valuable publications on coronaviruses from certain regions [34,35,36,37], a globally surveillance system for comprehensive genetic analysis of these circulating pathogens remains limited, with no genetic data on coronaviruses from Russia published to date.

In light of the above, this study aimed to develop a primer panel for the whole-genome amplification and downstream sequencing of all four types of human coronaviruses to investigate their circulation patterns and genetic diversity.

## 2. Materials and Methods

### 2.1. Specimens

A total of 14,704 nasopharyngeal swab samples were analyzed in this study. The specimens were collected from hospitalized patients with influenza and acute respiratory infection symptoms in St. Petersburg within the Global Influenza Hospital Surveillance Network (GIHSN) project [38] and were stored in the collection of the Smorodintsev Research Institute of Influenza (St. Petersburg, Russia). The specimens were collected from week 40 of 2017 to week 39 of 2023, inclusive. The number of samples collected by season is as follows: 2394 samples (16.28%) in 2017–2018; 1745 samples (11.87%) in 2018–2019; 2126 samples (14.46%) in 2019–2020; 790 samples (5.37%) in 2020–2021; 3668 samples (24.95%) in 2021–2022; and 3981 samples (27.07%) in 2022–2023. A total of 4031 (47.83%) samples were provided by female patients and 4397 (52.17%) samples by male patients. We also analyzed the 1979 hCoV-229E isolate from the collection of Smorodintsev Research Institute of Influenza (Russian Biobank of Influenza; https://rubin.influenza.spb.ru/en, accessed on 24 December 2024).

### 2.2. Isolation of Viral RNA

Nucleic acid extraction was performed using the NAmagp2000 RNA extraction kit (Biolabmix, Novosibirsk, Russia) and Auto-Pure96 automatic stations (Allsheng, Hangzhou, China). Some samples were processed using the column extraction method with the RUplus250 kit (Biolabmix, Novosibirsk, Russia).

### 2.3. Detection of the Respiratory Infection Pathogens

To identify pathogens, we used the CFX96 DNA thermocycler (Bio-Rad, Hercules, CA, USA) and the AmpliSens ARVI-screen-FL assay (CRIE, Moscow, Russia), following the manufacturer’s instructions. The samples were screened for the following respiratory viruses: B/C/E adenoviruses, bocaviruses, respiratory syncytial viruses, metapneumoviruses, rhinoviruses, type 1–4 parainfluenza viruses, and coronaviruses differentiated between the Alphacoronavirus and Betacoronavirus genera. Influenza A/B viruses were detected using the AmpliSens Influenza virus A/B-FL assay (CRIE, Moscow, Russia). SARS-CoV-2 RNA was detected using the S3102E SC2—Novel Coronavirus (2019-nCoV) Nucleic Acid Diagnostic Kit (Sansure Biotech, Changsha, China).

The species diversity of coronaviruses, belonging to the hCoV-NL63, 229E, HKU1, or OC43 species, was assessed using RT–PCR, targeting the most conserved regions of the N or ORF1ab genes, as developed by the US CDC: primers and probes for NL63 and HKU1 are listed in [39] and primers and probes for 229E and OC43 are listed in [40]. The BioMaster real-time RT-PCR kit (2×) was used to run the PCR assay. The amplification protocol was as follows: reverse transcription at 45 °C for 20 min and incubation at 95 °C for 5 min, followed by 44 cycles of denaturation at 95 °C for 15 sec and annealing at 58 °C for 1 min, with fluorescence detection during the annealing step.

### 2.4. Whole-Genome Amplification and Sequencing

Primers for whole-genome amplification of seasonal coronaviruses were designed in the PrimalScheme application (https://primalscheme.com/, accessed on 24 December 2024), according to the method described by Josh Quick [41]. To cover the whole genome, 900–1100 nucleotide amplicons were selected. Whole-genome amplification was performed using Biolabmix-Premium reagents (Biolabmix, Novosibirsk, Russia) and primers designed in PrimalScheme. Using 100 μM of each primer, we prepared equimolar primer pools. Pool 1 generated overlapping amplicons with odd numbers, while Pool 2 targeted amplicons with even numbers.

For each type of seasonal coronaviruses, we ran two separate multiplex PCR reactions (pools 1 and 2) using the one-step BioMaster RT-PCR–Premium kit (2×) (Biolabmix, Novosibirsk, Russia). Each reaction well contained 16 µL of the reaction mixture consisting of 10 µL of 2× buffer, 0.8 µL of enzymes, 0.4 µL of primers, 0.2 µL of SYBR Green, 4.6 µL of water, and 4 µL of the sample RNA. PCR amplification was performed under the following conditions: reverse transcription at 45 °C for 1 h, preliminary denaturation at 93 °C for 5 min, and then 44 cycles of denaturation at 93 °C (10 s), annealing at 57 °C (30 s), and elongation at 68 °C (4 min), finalized by elongation at 68 °C for 7 min. Amplification success was assessed by analyzing SYBR Green melting curve plots.

To prepare DNA libraries, the MGIEasy Fast PCR-Free FS Library Prep Set reagent kit (MGITech, Shenzhen, China) was used. Each reaction used 6 µL of whole-genome amplification products, containing from 35 to 200 ng of DNA. The library preparation protocol involved the enzymatic fragmentation of amplicons, end repair, A-tailing (adenylation), adapter ligation, and purification. Following purification, the libraries were pooled and subjected to rolling circle replication to amplify genomic DNA into DNA nanoballs. Sequencing with 10× target coverage was conducted on a DNBSEQ-G400 instrument (MGITech, Shenzhen, China) in 100 bp single-end read mode, using DNBSEQ-G400RS High-throughput Sequencing Set FCL SE100 kits.

### 2.5. Data Analysis

Reads were aligned to the reference sequence using the BWA-MEM 2. The reference sequence was chosen from a set of reference sequences representing various hCoV clades for each hCoV type. The best assembly was selected based on the following criteria: the percentage of reads mapped, the percentage of zero-covered positions, and the percentage of the genome covered at 20× depth. Samtools v. 1.19.2 software was used for BAM file processing. The consensus sequence was obtained using Unipro Ugene v45 software [42]. The MAFFT algorithm was employed to align the nucleotide sequences of HCoV-OC43, HCoV-NL63, HCoV-HKU1, and HCoV-229E (https://mafft.cbrc.jp/alignment/software/algorithms/, accessed on 24 December 2024). Phylogenetic trees were constructed using the maximum likelihood method (ML, GTR+G) in the RAxML program [43]. The resulting trees were visualized in FigTree and RStudio IDE using both the tidytree and tidyverse libraries. Coverage graphs were built using VizCov scripts (https://github.com/LMV-NIC-St-Petersburg/VizCoV, accessed on 24 December 2024).

## 3. Results

### 3.1. Species Diversity of Seasonal Human Coronaviruses Circulating in St. Petersburg from 2017 to 2023

Over the study period—from week 40 of 2017 to week 39 of 2023—we screened 14,704 samples. Of these, 5010 samples (34.07%) tested positive for viruses causing acute respiratory infections, and 424 samples (2.88%) were positive for human coronaviruses.

**Figure 1:**
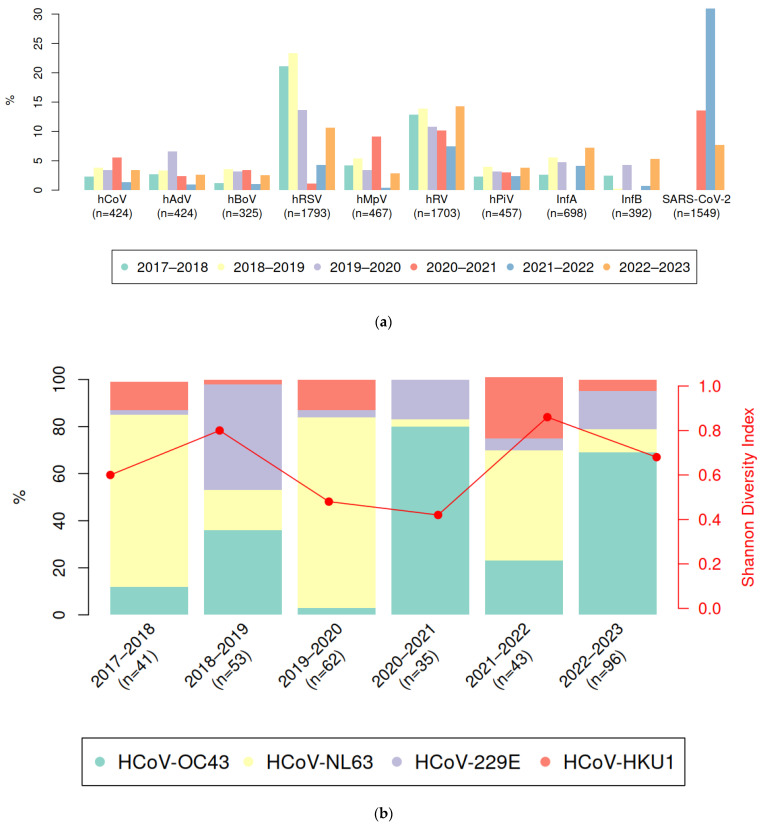
(**a**) Percentage of specimens testing positive for different respiratory viruses using RT-PCR (excluding mixed infections) over six consecutive epidemic seasons. (**b**) Assessment of the species diversity of seasonal coronaviruses in St. Petersburg in 2017–2023 (normalized Shannon index; from 0 to 1, where 1 is the maximum possible diversity); for details see Supplementary Table S1a–d.

The study revealed key patterns in the circulation of seasonal human coronaviruses. These viruses circulated annually and accounted for 8.46% of all viral acute respiratory infection cases. However, the prevalence of specific coronavirus species fluctuated seasonally, with different types prevailing in alternating patterns (Figure 1).

The variability in species diversity was assessed using the normalized Shannon diversity index, with seasonal values of 0.60, 0.80, 0.48, 0.42, 0.86, and 0.68, respectively. The 2021–2022 season showed the greatest diversity in circulating hCoV species, while 2020–2021 had the least diversity.

As Figure 1 illustrates, Alphacoronaviruses were more prevalent than Betacoronaviruses until the 2019–2020 season. This changed dramatically after 2020, which coincided with the onset of the COVID-19 pandemic caused by SARS-CoV-2, a member of the Betacoronavirus genus.

### 3.2. Primer Panel Design for Whole-Genome Amplification of Seasonal Human Coronaviruses

We used PrimalScheme to develop a primer panel for whole-genome amplification (WGA) of four seasonal human coronaviruses. The primer panel included 29–36 primer pairs (Appendix A) with a target amplicon length of 900–1100 bp; on average, the overlap between adjacent amplicons was 75–200 nucleotides.

The primer panels were validated using clinical specimens from hospitalized patients whose species variants were confirmed by RT-PCR. First, all primers were combined into stock solutions for pools 1 and 2 in equal proportions. Next, a temperature gradient PCR assay was run to determine optimal annealing temperatures. Melting curve analysis indicated two optimal values: 57 °C for HKU1/NL63 and 57.6 °C for OC43/229E.

The validation run identified all four target hCoV species: hCoV-229E/Russia/SPE-RII-28925S/2021, hCoV-229E/Russia/SPE-RII-25806S/2021, hCoV-229E/Russia/SPE-RII-1616/2019, hCoV-OC43/Russia/SPE-RII-2690S/2019, hCoV-HKU1/Russia/SPE-RII-15675S/2022, hCoV-NL63/Russia/SPE-RII-17546S/2022, and hCoV-229E/Russia/NVS-RII-MH71549S/2022. The length of the sequenced genomes ranged from 27,251 to 30,682 nucleotides. These genomes were annotated and deposited in the GenBank database (OR266952, OR266951, OR266950, OR266949, OR266948, OR266947, OR266946).

To further test the primer panel, we analyzed nasopharyngeal swabs collected from patients hospitalized in St. Petersburg clinics between 2017 and 2023. A total of 85 WGA products successfully passed the sequencing stage, demonstrating at least 70% coverage of the entire genome. The highest coverage was observed in samples with a Ct value below 25.

**Figure 2:**
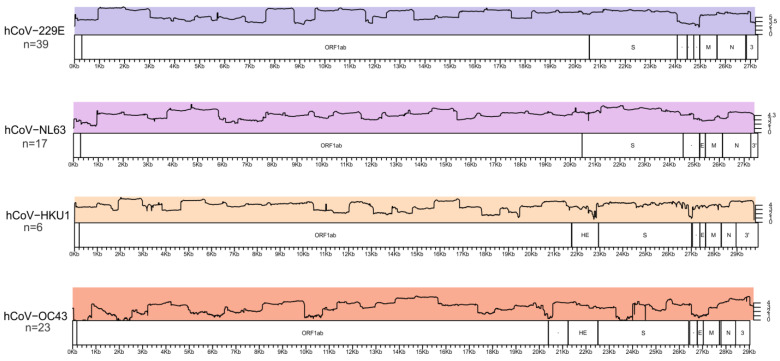
Average coverage plots for different hCoV species based on sequencing results. The number of specimens used for the calculations for each hCoV type is indicated in the figure.

As shown in the coverage plots (Figure 2), the developed primer panel covers the entire genome length and thus can be used for the genomic surveillance of seasonal human coronaviruses. For coverage plots of individual specimens, refer to Supplementary Figures S1–S4.

**Figure 3:**
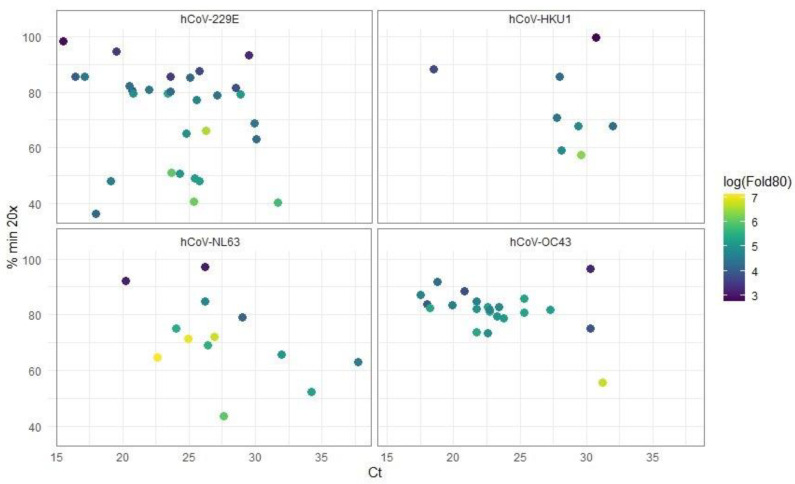
Scatterplots of percentage min 20× vs. Ct values for different hCoVs based on the sequencing results. The color gradient represents the log(Fold80) value. hCoV-229E: *n*=30, hCoV-HKU1: *n* = 8, hCoV-NL63: *n* = 14, hCoV-OC43: *n* = 22.

**Figure 4:**
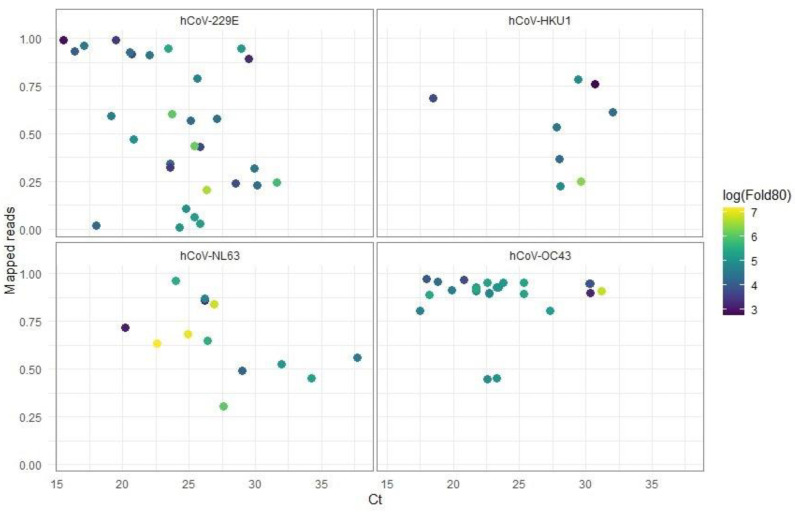
Scatterplots of the percentage of mapped reads vs. the Ct value for different hCoVs, based on the sequencing results. The color gradient represents the log(Fold80) value. hCoV-229E: *n* = 30, hCoV-HKU1: *n* = 8, hCoV-NL63: *n* = 14, hCoV-OC43: *n* = 22.

To assess the quality of the reference reads, we converted FASTQ files into BAM files using the Burrows–Wheeler aligner (BWA-MEM). For each virus species, the following metrics were calculated: total read count, number and percentage of mapped reads, mean and median coverage, percentage of nucleotides with at least 20× coverage, percentage of nucleotides with zero coverage, and Fold80, which describes how much additional sequencing is required to bring 80% of target bases to the mean coverage (Figure 3 and Figure 4).

As the above scatterplots illustrate, the percentage of genome coverage at 20× or higher and percentage of mapped reads has a weak dependence on the RT-PCR Ct values.

The total number of reads per sample ranged from 97,666 to 1,952,614 for hCoV-HKU1; 130,562 to 2,768,125 for hCoV-OC43; 249,260 to 3,273,881 for hCoV-229E; and 286,396 to 1,996,869 for hCoV-NL63. In terms of mapped reads, values ranged from 87,429 to 1,910,911 for hCoV-HKU1; 100,198 to 2,697,282 for hCoV-OC43; 201,307 to 2,281,985 for hCoV-229E; and 234,599 to 1,841,323 for hCoV-NL63 (Supplementary Figure S5). The minimum percentages of mapped reads were 22%, 48%, 31%, and 30% for hCoV-HKU1, hCoV-OC43, hCoV-229E, and hCoV-NL63, respectively. The maximum percentages of mapped reads were 98% for hCoV-HKU1, 99% for hCoV-229E, and 96% for both hCoV-NL63 and hCoV-OC43.

Since we did not normalize the libraries before pooling to simplify the sample preparation procedure, unequal DNA quantities resulted in varying raw data amounts between samples. This led to differences in coverage and the number of reads per sample.

### 3.3. WGS Analysis of Genetic Diversity of Seasonal Coronaviruses

**Figure 5:**
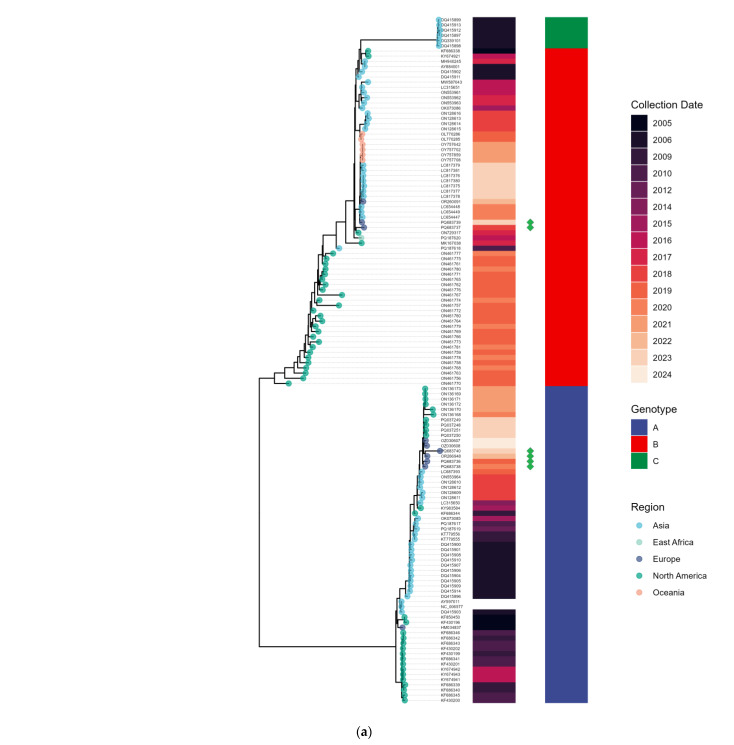

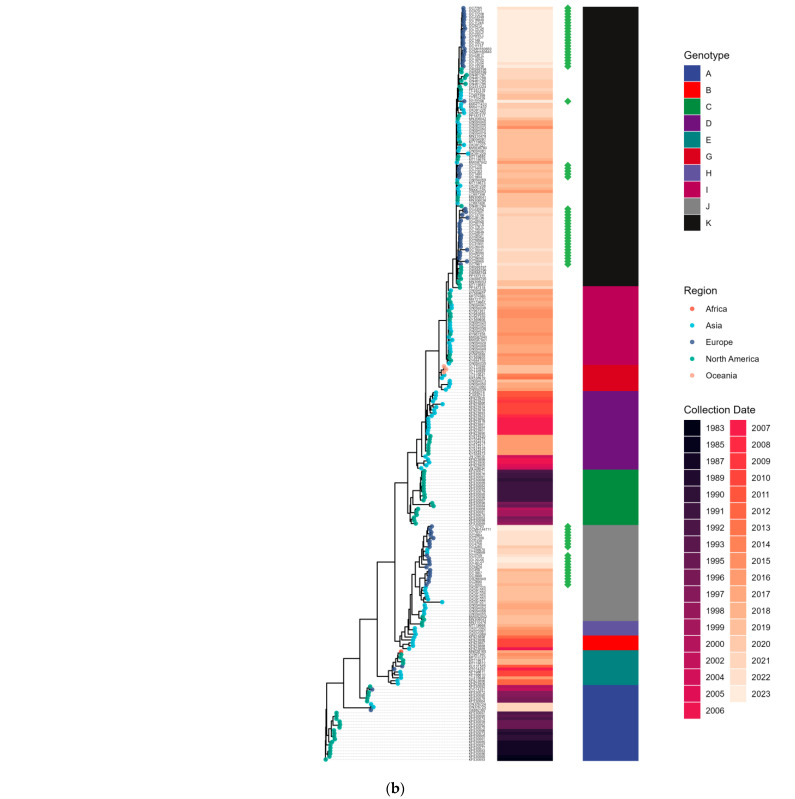

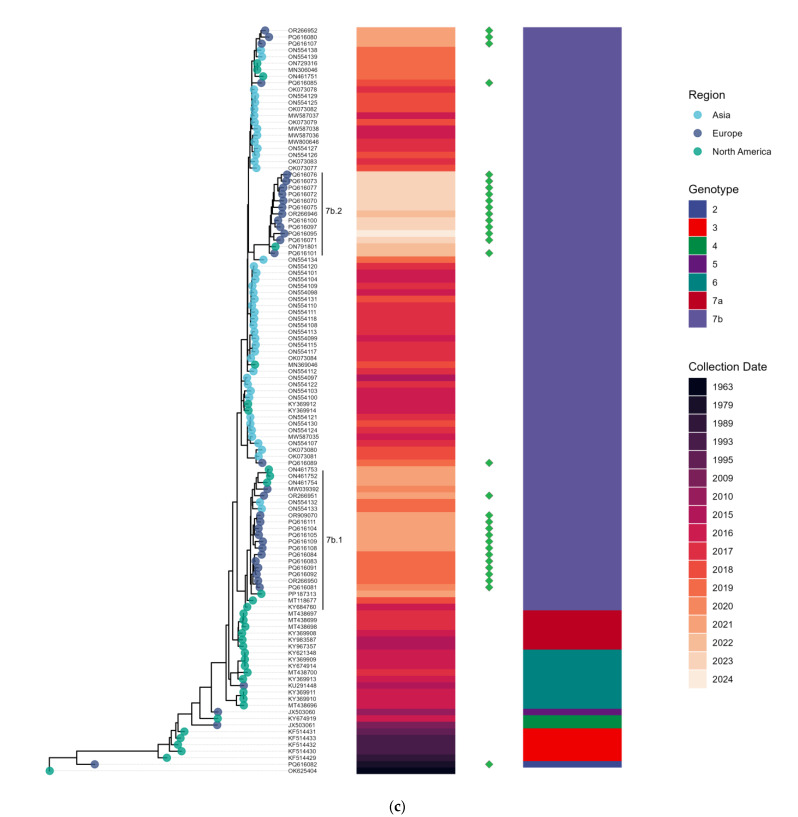

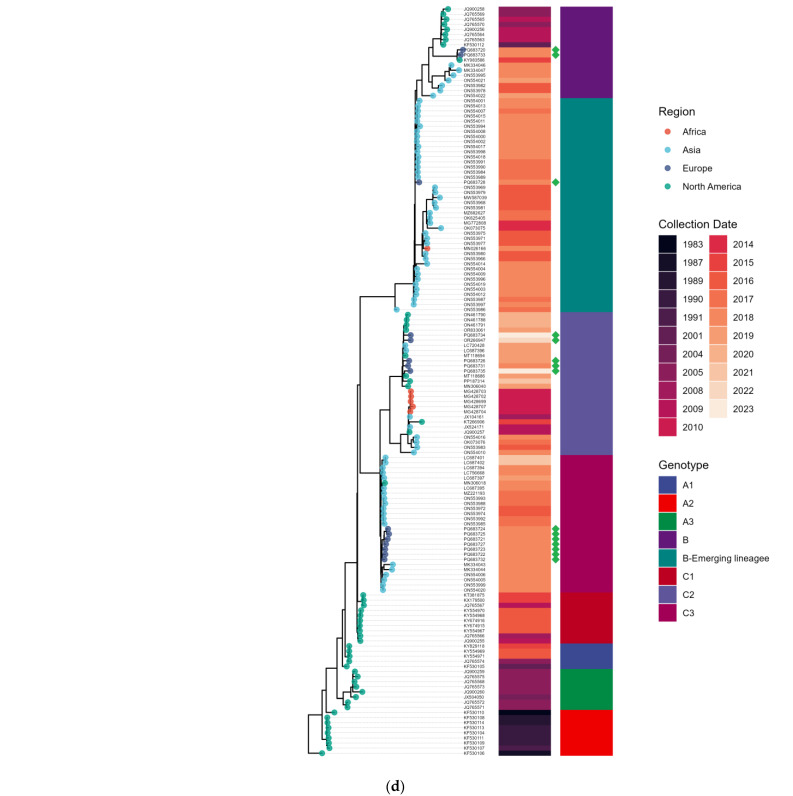
Phylogenetic trees of seasonal human coronaviruses circulating in St. Petersburg from 2017 to 2023. (**a**) hCoV-HKU1, (**b**) hCoV-OC43, (**c**) hCoV-229E, and (**d**) hCoV-NL63. Green diamonds indicate strains sequenced in this study.

Whole-genome sequences for 85 seasonal human coronaviruses with >70% genome coverage, including 23 hCoV-OC43, 6 hCoV-HKU1, 39 hCoV-229E, and 17 hCoV-NL63, have been obtained. The phylogenetic analysis of the sequenced viruses was conducted (Figure 5).

The sequenced Russian hCoV-HKU1 viruses represent both Genotype A and Genotype B. No evidence of the recombinant Genotype C was detected in this study (Figure 5a).

The majority of hCoV-OC43 specimens belong to Genotypes J and K, which were found to co-circulate during the period 2017–2024 (Figure 5b).

All sequenced Russian hCoV-229E viruses belong to Genotype 7b, which exhibits notable genetic diversity (Figure 5c). Within Genotype 7b, two distinct subclades can be clearly delineated: 7b.1 (hCoV-229E/Russia/SPE-RII-1616/2019-like viruses) and 7b.2 (hCoV-229E/Russia/NVS-RII-MH71549S/2022-like viruses). Subclade 7b.1 viruses exhibit six amino acid substitutions in the Spike protein (A41D, V288E, Y305H, N307K, R311G, N845S), and subclade 7b.2 viruses harbor ten non-synonymous substitutions in the Spike protein (T19I, A41D, R102T, N213D, V288E, Y305H, F350Y, N406G, L886M.

We also sequenced a historical hCoV-229E isolate collected in the USSR in 1979 (hCoV-229E/USSR/Leningrad-RII-MH144219V/1979). Phylogenetic analysis revealed that it belongs to Genotype 2, which aligns with the estimated timeframe for Genotype 2 circulation (1970–1980) based on Bayesian reconstruction [44]. To our knowledge, this represents the oldest sequenced hCoV-229E isolate from Eurasia. Thus, the primer panel for hCoV-229E demonstrates robust performance across a wide range of viruses, including the historical Genotype 2 isolate.

The phylogenetic analysis of hCoV-NL63 specimens collected in St. Petersburg identified Genotypes B, C2, and C3 (Figure 5d). As described in [45], the C2 and C3 genotypes differ from A1–A3 and B by an I507L substitution in the receptor-binding domain (RBD) of the S protein. The majority of hCoV-NL63 specimens belong to Genotype C (C2 and C3). Notably, McClure et al. [46] proposed splitting Genotype B into two subgenotypes, B1 and B2. In our study, two out of three Genotype B specimens were classified as B2.

## 4. Discussion

The PubMed database [https://pubmed.ncbi.nlm.nih.gov/] indexes over 120 times fewer publications on seasonal coronaviruses than on COVID-19 and SARS-CoV-2, with the latter exceeding 200,000 articles. This is especially noteworthy since seasonal coronaviruses were discovered nearly 60 years ago, while SARS-CoV-2 only emerged at the end of 2019. These figures signal that many countries, including Russia, do not effectively and comprehensively monitor the dynamics of coronavirus infection across epidemic seasons.

Interest in the evolution of coronaviruses has grown in recent years. Many publications now describe retrospective studies of hCoV genetic diversity, reporting new genotypes of these pathogens. As of January 2024, the GenBank collection includes 4267 sequences of seasonal coronaviruses, reflecting exponential growth in the collection of genetic data. Experiments to identify animal reservoirs of pandemic coronaviruses have highlighted their strong evolutionary potential, broad host range, and substantial genetic diversity [45,47].

Our study aimed to develop whole-genome sequencing primer panels for four human seasonal coronaviruses to facilitate the genetic monitoring of these pathogens. Primer design inherently involves balancing competing priorities: shorter amplicons offer better sensitivity and are less affected by RNA degradation in samples, but they increase the complexity of the primer pool and the likelihood of off-target effects [48]. Additionally, the identification of conserved primer-binding sites in an evolving viral genome further complicates this process. In this study, we designed four primer panels generating amplicons of 900–1100 bp in length. This design closely aligns with the amplicon length (~1200 bp) deemed optimal for the “Vivaldi” panel, developed for seasonal hCoV sequencing [46]. However, it is worth noting that the primer sequences for the Vivaldi panel were not disclosed at the time of this publication, limiting direct comparisons and broader reproducibility.

This study has some limitations, including low geographic representativeness (most of the specimens were collected in St. Petersburg) and a relatively small number of specimens. However, it is important to note that we covered multiple epidemic seasons and successfully sequenced genetically diverse viruses representing different genotypes and genetic clades of seasonal hCoVs.

Additionally, the primer panels developed in this study demonstrated uneven genome coverage. In particular, several primer pairs targeting the Spike (S) region of hCoV-OC43 showed suboptimal performance. As with SARS-CoV-2 primer panels, periodic updates to the primers may be required in the future to adapt to viral evolution and improve overall performance.

Using these custom primer panels, we analyzed the species and genetic diversity of seasonal human coronaviruses that circulated in St. Petersburg from 2017 to 2023. We report the first near-complete genomes of seasonal human coronaviruses (hCoVs) from the Russian Federation, addressing a gap in the genomic epidemiology of these viruses. Historically, Europe has been underrepresented in studies of seasonal hCoVs, with the majority of genomic data originating from North America (USA) and Asia (China).

The predominant type of seasonal coronavirus varied across epidemic seasons. However, our dataset did not provide any clear evidence to suggest that the prevalence of seasonal hCoV species was influenced by the COVID-19 pandemic. However, additional genomic data are needed to draw definitive conclusions regarding the influence of the COVID-19 pandemic on the genetic clade distribution of seasonal hCoVs. It is well established that non-pharmaceutical interventions (NPIs) significantly altered influenza circulation patterns and may led to the apparent extinction of the influenza B/Yamagata lineage [49]. Studies conducted in French and Dutch cohorts have clearly demonstrated that the NPIs implemented during the COVID-19 pandemic likely contributed to the waning of seasonal hCoV-specific antibodies [50,51]. The subsequent relaxation of these restrictions was associated with the reemergence of seasonal hCoVs [52,53]. However, the impact of unevenly implemented NPIs—ranging from strict measures in regions like China and the EU to more lenient approaches, such as those in the Russian Federation—on the genetic diversity and evolution of human seasonal hCoVs remains an open question and warrants further investigation.

Seasonal coronaviruses demonstrate broad genetic diversity, with each of the four species comprising several genotypes. The hCoV-OC43 viruses circulating in St. Petersburg over the past six years predominantly belong to the K and J genotypes. It is noteworthy that the K lineage actively evolves, accumulating numerous substitutions in the S gene. hCoV-HKU1 viruses were grouped into two genotypes, A and B, while hCoV-NL63 included B, C2, and C3 lineages.

All sequenced Russian hCoV-229E viruses belong to Genotype 7b, which demonstrates significant genetic diversity. Within Genotype 7b, two distinct subclades can be clearly identified: 7b.1 or hCoV-229E/Russia/SPE-RII-1616/2019-like viruses; these viruses carry six amino acid substitutions in the Spike protein (A41D, V288E, Y305H, N307K, R311G, N845S) and 7b.2 or hCoV-229E/Russia/NVS-RII-MH71549S/2022-like viruses. These viruses harbor ten non-synonymous substitutions in the Spike protein (T19I, A41D, R102T, N213D, V288E, Y305H, F350Y, N406G, L886M, I971V).

These findings align with the phylogenetic analysis presented by McClure et al. [46]. Several of these mutations may have functional significance. For instance, R311G affects receptor-binding loop 1, while N406G impacts receptor-binding loop 3. Furthermore, residues 307 and 350 are considered supporting residues, which could influence receptor interaction [54]. Previous studies have shown that HCoV-229E viruses circulating between the 1960s and 2015 can be divided into six distinct classes based on the amino acid sequence of their receptor-binding domain (RBD) [55]. These classes exhibit antigenic differences, with experimental evidence demonstrating a progressive increase in host receptor (human aminopeptidase N, hAPN) binding affinity from Class I (corresponding to genotype 1) to Class VI (corresponding to genotype 6). The numerous substitutions observed in the Spike protein of hCoV-229E genotype 7 viruses, including some affecting the receptor-binding domain (RBD), underscore the need for phenotypic characterization of genotype 7 viruses to better understand their functional and antigenic properties.

Based on our genetic observations, it is reasonable to speculate that viruses within subclades 7b.1 and 7b.2 may exhibit differences in their antigenic properties and/or receptor-binding characteristics. However, further studies are required to confirm these hypotheses and evaluate their implications for viral evolution and immune escape. Notably, subclade 7b.2 (hCoV-229E/Russia/NVS-RII-MH71549S/2022-like) viruses were found to be circulating in Russia between 2022 and 2024, underscoring their ongoing relevance in the region.

Thus, our study on the development of a primer panel not only expands the methodological toolkit for whole-genome sequencing of seasonal hCoVs but also provides insights into the genomic epidemiology of acute respiratory infections caused by seasonal coronaviruses in St. Petersburg in 2017–2023.

## 5. Patents

Patent pending. Application no. 2024128521 (Russian Patent Office).

## Data Availability

Genomic sequences have been submitted to the GenBank database under the following accession numbers, namely PQ616070–PQ616112 (hCoV-229E), PQ683720–PQ683735 (hCoV-NL63), PQ683736–PQ683740 (hCoV-HKU1), and OR266949, with 17 additional sequences currently in progress (hCoV-OC43). For additional information see Supplementary Table S2a–d.

## Supplementary Materials

The following supporting information can be downloaded at: https://www.mdpi.com/article/10.3390/v17010013/s1. Table S1a: Distribution of hCoV-typed samples by epidemic seasons. Table S1b: Distribution of hCoV-typed samples by patient gender. Table S1c: Distribution of hCoV-typed samples by age groups. Table S1d: Shannon diversity index for all hCoV-typed samples by epidemic seasons. Table S2a: The GenBank accession numbers for the Russian hCoV-229E virus genomes analyzed in this study; Table S2b: The GenBank accession numbers for the Russian hCoV-NL63 virus genomes analyzed in this study; Table S2c: The GenBank accession numbers for the Russian hCoV-HKU1 virus genomes analyzed in this study; Table S2d: The GenBank accession numbers for the Russian hCoV-OC43 virus genomes analyzed in this study; Supplementary Figure S1. Coverage plots of six hCoV-HKU1 genomes generated using the VizCoV script; Supplementary Figure S2. Coverage plots of 10 hCoV-NL63 genomes generated using the VizCoV script; Supplementary Figure S3. Coverage plots of 10 hCoV-229E genomes generated using the VizCoV script; Supplementary Figure S4. Coverage plots of six hCoV-OC43 genomes generated using the VizCoV script; Supplementary Figure S5. Number of reads aligned to the reference genome by hCoV type.

## Appendix A

**Table A1:** Primers for amplicon sequencing of seasonal human coronaviruses.

Name	Primers	5′-Position *	GC-Content	Tm
OC43_F1	ATCTCTTGTTAGATCTTTTTGTA	26	26	53.9
OC43_R1	AAGCCYCTARGCCATNTGCAAG	998	50.00	60.87
OC43_F2	TATGACCAGGTGCATGATGARC	872	50.00	60.1
OC43_R2	CGCATATTAAAATAATCAAT	1877	36.00	59.86
OC43_F3	TGTRGCAATTTAYAYCAACG	1777	43.7	56.4
OC43_R3	GCACAAGGAANTCTCCATGCT	2739	50.00	61.32
OC43_F4	CTGAACCACCTAAAGTYGYAGNT	2625	50.00	60.21
OC43_R4	TATGCAANACACTGCCAATCAC	3630	47.83	61.16
OC43_F5	GRRTCTTTGGNTNTTATACAAGCAAC	3496	42.31	61.06
OC43_R5	CTTAGTGCCCTCAACAGCAGTT	4504	50.00	60.99
OC43_F6	AGTGTGCCTTCTGATGTGTCYTT	4394	43.48	60.69
OC43_R6	CAACTTGACTAAACACAACACGCA	5394	41.67	60.98
OC43_F7	GAGGCATGGYTTGAATTTCGT	5287	45.45	61.06
OC43_R7	ACATCATCNACTTTTAAAAC	6260	50.00	61.05
OC43_F8	GCCATGAGAAAGCTTCYCTCA	6171	50.00	61.06
OC43_R8	ACTATCAATTCAATGACAA	7151	52.17	61.35
OC43_F9	GCATGYCAGTTCTGCYTGGCA	7019	52.38	61.85
OC43_R9	TGTTTTATAGAAGAATGCGCAGTTG	8010	44.00	60.88
OC43_F10	CTACTGCTAAYACTGGTACNTCTG	7899	50.00	60.99
OC43_R10	GCAGAGGAAAGCACACARCCACTAGC	8898	52.38	61.52
OC43_F11	TGGCTCTACAGTGTTTAATRTCCC	8752	45.83	60.89
OC43_R11	GCAACATCAGACAAAGAATTCTTAAGC	9736	37.04	60.42
OC43_F12	GTYCGTAGTRATGGYACATTTGAAG	9644	43.48	60.69
OC43_R12	GAGCCCANACRTTAAAATCTTCTACAG	10,623	40.74	60.90
OC43_F13	GAYGCTCARGTTGTTCARTTGC	10,505	50.00	60.53
OC43_R13	GAAAACAARCCCCAATAACAGC	11,511	47.83	61.12
OC43_F14	GTGGGTTGCTGTGAATGTYTTG	11,422	50.00	60.47
OC43_R14	TTATCAACTAYCTGGTCATAAACAC	12,393	37.93	61.02
OC43_F15	TGGTGCGTAAGTTAGATAATCAAGCT	12,267	38.46	61.07
OC43_R15	CGCCAAAATCCACAAACTCNACAAA	13,251	50.00	61.09
OC43_F16	CAGGANTCATATGGTGGTGCRT	13,097	50.00	60.93
OC43_R16	GCATTATTCACATACAATTCRCAATCC	14,067	37.04	60.63
OC43_F17	ATGTTATTGCAGCCCCAGGATG	13,979	50.00	61.20
OC43_R17	CAACAGTGCGGGCTCTATTCTT	14,953	50.00	61.12
OC43_F18	AAAGCYAGRCTCTATTATGAAGCA	14,833	41.67	60.71
OC43_R18	CATCCAGCTCCTAATATACGACTAGG	15,802	46.15	60.40
OC43_F19	CCAAATGTTGGGTTGARCATGACA	15,692	41.67	60.76
OC43_R19	AACATACTCACCTAAAACTGTCTTACC	16,668	35.71	60.98
OC43_F20	CGCGAATTGATTCTCTCTTGG	16,578	50.00	60.46
OC43_R20	CTTTCATGTGTTGTAACGCCCTT	17,524	43.48	60.25
OC43_F21	GTTGATACAGTGTCYGCCTTRG	17,436	50.00	61.17
OC43_R21	GGTCTAACAACATCCCAGCGAT	18,374	50.00	60.60
OC43_F22	GAAGCCACTGGTTTGTTTGCTG	18,262	50.00	61.23
OC43_R22	CCATCCATATACACACAAGRCGTAT	19,237	47.83	61.00
OC43_F23	TCTTCCYGGCTGTAATGGNGGT	19,119	50.00	61.02
OC43_R23	CCGACATTACCGGGTTTACCAT	20,084	50.00	60.60
OC43_F24	TGCTGTGCGTAAAGAAGGTNA	19,989	50.00	61.30
OC43_R24	TCAAAGGGTAARGTTATACAATCCCCA	20,931	37.04	60.76
OC43_F25	AAAAAGGAGTAGCNCCRGGTTC	20,819	50.00	60.99
OC43_R25	CCTTTAACATCTAAAACTATGCAT	21,804	41.67	60.64
OC43_F26	GTGTTGATGTCGCTATTCAAGAAGTT	21,692	38.46	60.78
OC43_R26	TACCAGNACGGTTGCATTTAAAGGC	22,680	47.83	61.41
OC43_F27	TCTAAAGCTGGCAACTCCATTTT	22,571	43.48	60.76
OC43_R27	TACAATNGCAACGCCCAAAAGAAT	23,541	45.45	61.10
OC43_F28	TTAGCAGTGTYTGGCCTC	23,424	50.00	60.80
OC43_R28	CAAGTCAGAGGCATGACATA	24,394	42.31	60.23
OC43_F29	TCAAGAAGGTGGTACYTTTTATGCAT	24,292	34.62	59.72
OC43_R29	GAACAGTGCTCACCTATRCCAAC	25,278	50.00	61.06
OC43_F30	TAGGYACTTGYCCTGCAGGTACT	25,169	50.00	61.34
OC43_R30	CACAGAAACTACCATATTCAACCAACTG	26,129	39.29	61.32
OC43_F31	CAGANTTTACTATAGGTAATATGG	26,022	36.67	60.69
OC43_R31	TCCTAGATGATTGGRTTTTGACACA	26,987	40.00	60.90
OC43_F32	GTCGTCTYACCGCTCTTAATGCT	26,889	47.83	60.98
OC43_R32	CATCTTCTAATTCTGAGACATTAAAACCGT	27,834	33.33	61.02
OC43_F33	GACTAAGTTCGTCTTTGATT	27,703	40.00	60.94
OC43_R33	TCTGGGTTRAAACTCCAAAAACT	28,709	44.00	61.29
OC43_F34	GCATTGAATAAYGTNTATYTTGG	28,603	42.31	61.71
OC43_R34	TAGTCGGAATAGCCTCATCGCT	29,579	50.00	61.00
OC43_F35	GGACCGCATGCTAAAGACCA	29,463	50.00	61.12
OC43_R35	CACCAGATRCCGACATAAGGTT	30,432	50.00	60.86
OC43_F36	AGGATCGCGTAGTAGAGCCA	29,710	50.00	61.26
OC43_R36	GCCATAATTAACTTCATTCATTTA	30,693	36.00	60.78
**Name**	**Primers**	**5′-Position**	**GC-Content**	**Tm**
229E_F1	ACTTTGTGTCTACTyTTCTNAACTAAACG	46	34.48	61.21
229E_R1	ACAACACACAGTTTATTACTGAT	1119	30	56.72
229E_F2	AATGGTTCCAACATCCTAGAGGC	968	47.83	60.63
229E_R2	CAATGGTTTATAGACTGCAG	2068	38.46	60.79
229E_F3	TGCTGTACTTACAATCGCCAAC	1915	50.00	60.40
229E_R3	AGCTTGACTCTGTAAACAGG	3048	45.83	60.87
229E_F4	CAGTAATAGATGAGCACGTCTT	2886	45.83	61.06
229E_R4	GCATCATTTGTCCAGGGCTGA	3987	52.38	61.06
229E_F5	TGCTCATCAATTTTTCGTGGTGC	3869	43.48	61.11
229E_R5	GTTTGTCCTTTTCGGATGGCAC	4937	50.00	60.78
229E_F6	GTGGTGTGCTTAGTGyTGCTAT	4806	50.00	61.12
229E_R6	ACCTTTGTCAACAGGTCCAGAAA	5878	43.48	60.57
229E_F7	GCAAGTTGGTTATTGTRTCCATGG	5716	45.83	61.17
229E_R7	AACGTTGCACACCATTRACAA	6836	45.45	60.66
229E_F8	TGTGATGAACTGyTTGTCACTGT	6680	43.48	60.94
229E_R8	ACACACNARACCACACAGTA	7798	50.00	60.92
229E_F9	GCTAAGGGTTTGACTTTCTTGTTGAC	7655	42.31	61.38
229E_R9	GCACACCAAATCCATGCATA	8784	50.00	60.98
229E_F10	CGCATGTTTGGTNACCTTTCTG	8621	50.00	60.78
229E_R10	AACCACCATACATAACACCRTCAAA	9737	44.00	60.83
229E_F11	TGGTTCYCCTGGCTAYAATCT	9619	52.38	60.95
229E_R11	CAACAGCACAGTTTTAACACCATCA	10,696	40.00	61.06
229E_F12	TGCTGGTTATGCTACTTTGTGCA	10,560	43.48	61.31
229E_R12	TCCAAACAACACCAGCGTAG	11,642	50.00	61.50
229E_F13	CCTCTTTCCGTTATCCCTGC	11,525	50.00	60.75
229E_R13	CGTCAAATRCACGGACACAGTA	12,598	50.00	61.61
229E_F14	TGTACATGTGACCGGACTGCTA	12,470	50.00	61.33
229E_R14	GAATGRGTRTTAACATCTTTGTTCCAC	13,568	35.71	61.02
229E_F15	TCCAAACACRGCTTTTGGACCT	13,443	50.00	60.80
229E_R15	GGCTGTAGTTGCRTCACCAGAA	14,544	50.00	61.06
229E_F16	TGTCACATGTTGTACGGCTAGTG	14,406	47.83	61.11
229E_R16	GGGAATGAAAGATGTGGTTTGTG	15,521	45.83	60.88
229E_F17	GTGCGCNTATGATCAYGTGTTTG	15,360	47.83	61.04
229E_R17	TGAGGATCACCAACATATACAATGTGT	16,487	37.04	61.08
229E_F18	GCGTTACCTGAAGTTAATGCAGAC	16,363	45.83	60.75
229E_R18	CACTGCCAGTGTTTGTTARAACA	17,461	43.48	59.87
229E_F19	GCGTCATGTCAGAGGTTGGTTA	17,307	50.00	60.79
229E_R19	ACAACCTCACATGNACCGTC	18,410	52.38	60.97
229E_F20	ACATTGAAYCTYGAAGGTGT	18,268	47.83	61.01
229E_R20	TCAACAAGTGTAAACCGCCTAGAG	19,378	45.83	61.12
229E_F21	CTCGNAGCACAATGGAAGAAGA	19,238	50.00	61.37
229E_R21	GTCCTTGTGCGAAATCACCAAG	20,317	50.00	60.53
229E_F22	CGGAGGTTCRATTGCTATTAAAGTAAC	20,151	40.74	61.00
229E_R22	GTCAGCATAAGAAGCYAAYGCAAC	21,286	45.83	61.04
229E_F23	GCACTACCTAAGACAGTTCGTGAG	21,122	50.00	61.22
229E_R23	CATCTGCACAAACGCCAAAACT	22,244	45.45	60.98
229E_F24	GCAAGCTGTTGTTGGTGCTATG	22,090	50.00	61.10
229E_R24	AGCCARTCTACCAGTAATCAGCC	23,230	47.83	60.44
229E_F25	CCATTTAACNTCTCAGTTGAGGCAG	23,104	42.31	61.00
229E_R25	CCTGTCGTGAAAYTTCAGCACT	24,181	50.00	61.24
229E_F26	CGACGTTGAAAAGATYCACATACAG	24,064	44.00	60.82
229E_R26	GTGGCCAAAGRAGCCACAGTAC	25,163	50.00	60.21
229E_F27	GACAATTGTACGGGTGACATTGTC	25,004	45.83	60.69
229E_R27	CAACAGTAACACCATTTGGGAGC	26,103	50.00	60.67
229E_F28	GGGTTGCTGTTGATGGTGCTA	25,990	52.38	60.99
229E_R28	GATCNTTGTCAAGCCAAAGCAAG	27,063	47.83	60.86
229E_F29	AATCCTTCAANTGACAGAAACCA	26,190	47.83	60.63
229E_R29	TCGAAACCGTTCCATTAGCAGTT	27,266	43.48	61.06
**Name**	**Primers**	**5′-Position**	**GC-Content**	**Tm**
HKU1_F1	ACGTTCGTACCGTCTATCAGCT	15	50.00	61.43
HKU1_R1	ACATCTTCTGTAGGTTGTGCAAC	1095	41.67	61.01
HKU1_F2	AAGCAGTCTGTATGGCTTGCC	976	52.38	61.06
HKU1_R2	ACCCAATAACTTAAATTGAGTAACCAACC	2027	34.48	61.08
HKU1_F3	TGTCTCAATTTTCACAAGAAGTTTCTGA	1922	32.14	60.66
HKU1_R3	ACGTCTTCAACATCCTCAATAG	2978	45.83	60.99
HKU1_F4	GTGGTTGGTTATCAAGTTYGTGC	2848	47.83	60.86
HKU1_R4	AACCACATTTTAAACAACACCAATTAGC	3904	32.14	60.76
HKU1_F5	AAGGTGGGTTATAATCAAAGNTTTGTTGA	3772	31.15	60.98
HKU1_R5	TGAAACAAAACAATCATCACCACTCA	4856	34.62	60.57
HKU1_F6	CAAATTACTTCAGTTGTTGGTACTAAAGC	4756	34.48	60.47
HKU1_R6	ACCTGCACAATTACAGCCAAT	5801	43.48	61.07
HKU1_F7	TCAGGTGCANTYTGTGARTTAGAACTT	5677	33.33	60.70
HKU1_R7	CTGTNGGTGATTTAACTAGGCGTG	6746	45.83	60.46
HKU1_F8	GGTTAAGTTAAARGGTGTTARAAAGACTG	6582	37.93	61.07
HKU1_R8	ACACCACCAACAATAGTACTACACTT	7696	38.46	60.63
HKU1_F9	CCTGCTTTTGTYTTGTTGCGG	7558	52.38	60.97
HKU1_R9	AATAGGRACACTTGCCTCTTGC	8667	50.00	60.80
HKU1_F10	TGTACAGGGTAATGTTGCTAAGG	8523	45.83	61.19
HKU1_R10	CTAGTATAATCTCCAAAAGCACGC	9630	42.31	60.72
HKU1_F11	GCCTGGAACTTTTTGTGGTAGAGA	9468	45.83	61.31
HKU1_R11	GTCGGCCATTATAYGCAGCTAA	10,549	50.00	61.04
HKU1_F12	TATGAAYGAACCTGATTATTCTGC	10,352	41.67	60.65
HKU1_R12	ACATAAGTAAAATTCACAGCAGG	11,408	35	58.82
HKU1_F13	GCTGGTGTTAAATTGCAATCTAAAACAA	11,098	32.14	60.80
HKU1_R13	CAAAGCCTGCAAAACRGTACTATC	12,226	45.83	60.98
HKU1_F14	TAGCTTTTGATAAGCTTGCTCAA	12,103	35.71	60.91
HKU1_R14	TACTCAGTAGCAACACCAGCCT	13,163	50.00	61.27
HKU1_F15	GTTGTTTTAGAGCTTGATCCTCCTTG	13,018	42.31	60.78
HKU1_R15	TCTGCAAAANTGACAGTATTAAG	14,140	38.46	58.90
HKU1_F16	GTGTGAAATTCTTTGYGAGTATGCTGA	14,000	37.04	61.01
HKU1_R16	TCAAATGATAATGCCTCATA	15,110	30	50.02
HKU1_F17	ACTCAAGATGGTAATGCTGCAATTAC	14,889	38.46	60.62
HKU1_R17	GAACARAATTCATGAGGACCA	15,983	41.67	57
HKU1_F18	GTGATGATGGTGTYGTYTGTTATAACTC	15,838	39.29	60.96
HKU1_R18	ACTCACCTAAAACTGTCTTACCAGTAC	16,924	40.74	60.96
HKU1_F19	GCTATGCTTCTGCTACYATTCAAG	16,801	45.83	60.34
HKU1_R19	ACGCTTAGCAACATARTTCTGAC	17,905	38.46	61.05
HKU1_F20	GGGACAGACAACACATGAGAGTT	17,786	47.83	60.75
HKU1_R20	CACAAGTATAACTATGGCGCCAAC	18,841	45.83	60.57
HKU1_F21	TGGTCTGCCAGTTTTGAACTTAC	18,720	41.67	60.95
HKU1_R21	CAACTTTGTCATTNATAATAGCRCAAGG	19,797	35.71	60.29
HKU1_F22	GGTGGNGCTGTTTGTTCNAAGCA	19,587	50.00	60.98
HKU1_R22	CTGTACAAACACTCTTACTACTACCACA	20,667	39.29	61.02
HKU1_F23	GGAGGTTTGCAYTTGCTTATAGG	20,550	45.83	61.05
HKU1_R23	GCAGTGCCAGCCAATTTCAAAG	21,604	50.00	61.62
HKU1_F24	TGAAATAGATGGCAATGTTATGC	21,488	34.48	61.18
HKU1_R24	GTTCCAYCTAGAATCAACAAC	22,552	50.00	60.59
HKU1_F25	ATTTCTCTTAAAYCNGGTTCTTAT	22,459	45.45	61.07
HKU1_R25	CCACGTTCTTGATAAAAATGAAAATACA	23,533	31.16	60.71
HKU1_F26	ATTACAGCTTGTCARTAYACTATGTG	23,379	37.93	61.06
HKU1_R26	TCAGGTARACAAGAACAYCTACACCA	24,462	45.00	66.93
HKU1_F27	TGTCCTRYYGGTACTAANTATCGT	24,366	36.00	60.49
HKU1_R27	ACAATAARTCATGRCAAGCNGCATA	25,419	45.45	60.01
HKU1_F28	GAACCYTTTAATGTYAGTTTTGTTA	25,284	47.83	61.02
HKU1_R28	CAAACCATAAGGAGCATTTTGAAC	26,361	38.46	61.23
HKU1_F29	GAGAARGTTAATGAGTGTGTTA	26,260	40.74	60.53
HKU1_R29	CAACTAAYYTAGANACAGCCTCA	27,300	42.31	60.79
HKU1_F30	TGTATTGTACCTTTAAAYGTNTGGTGTC	27,186	45.45	61.24
HKU1_R30	CAAGGCAGTATCCATACCACTA	28,268	50.00	60.58
HKU1_F31	TATGTTACTGTAGCTAAGGTGCAAGT	28,129	46.15	61.28
HKU1_R31	GCACCTGGTGTAGGRGCTAATTC	29,250	45.83	60.94
HKU1_F32	ACTTCTAYTCCCTCCGATRTTTCG	28,783	50.00	61.06
HKU1_R32	AGCYCTTCCCGGAGGCGTTTATACTAC	29,842	47.83	60.44
**Name**	**Primers**	**5′-Position**	**GC-Content**	**Tm**
NL63_F1	CTAGTGCTGTCATTTGTTATG	46	39.29	60.85
NL63_R1	ACCAGGAACRACATTACCAGGA	1110	50.00	61.27
NL63_F2	TGTTAAATGCAATTGTGGTTCTGAGA	1011	37.04	61.22
NL63_R2	AAACCATCAAGCTTAAAACCACTAAACT	2075	32.14	60.92
NL63_F3	TTGTGYACCTGTAGTTTGCCCTAAAG	1930	50.00	59.69
NL63_R3	TGAGYTTGACTTTATGGGTAGGTTC	3022	44.00	60.96
NL63_F4	GATTATCTTTGGTATGTTGTAGATG	2887	34.48	60.93
NL63_R4	CAAAGACACCAAAACCATCAATAGCT	3951	38.46	60.96
NL63_F5	TGTTTGTACAGGAYCCANCACC	3827	50.00	60.73
NL63_R5	GCAGGAACATTGGCAATAACACA	4903	43.48	60.56
NL63_F6	GAGAGTTGTACCATTTATATGTGTGTTGT	4801	34.48	60.72
NL63_R6	CACAACCACCTACTACAACAATAGC	5878	44.00	60.25
NL63_F7	TCAGGTTCYTTTGATAACGGTCACT	5770	40.00	60.84
NL63_R7	ACCAGGNCCANANGAATCACAA	6828	45.45	60.88
NL63_F8	TGCTGACTGYGTAGCTTGTTCT	6684	45.45	60.08
NL63_R8	GCTTCAAACACCTTCAACTGACC	7807	47.83	60.74
NL63_F9	TGGTATGTATGTTTATTTGTTGTTGC	7696	34.48	60.96
NL63_R9	TGCAGCAAAACYAAACCATRTGAG	8743	41.67	60.82
NL63_F10	TGGTGTTTTTACTGTTGTTTGTGCA	8559	36.00	61.12
NL63_R10	GCACTCTCAACTTGCAAACT	9670	45.45	61.13
NL63_F11	GAGCTTGTGGTTCTCCTGGTTA	9527	50.00	60.14
NL63_R11	CCTATTGAACCAATACAAAATGCCCC	10,652	42.31	61.35
NL63_F12	GGTGCCATTGTTTTTAGGTT	10,524	41.67	61.05
NL63_R12	GAGGCCATGTCAAAGTTTCAACA	11,625	43.48	60.18
NL63_F13	GTGATGGTTCTGTTCATTATGCTGG	11,525	44.00	60.83
NL63_R13	AACCGACTTAGTACACCTCTTTATAACA	12,605	35.71	60.61
NL63_F14	ATGGCACGGACATYGATAAGTG	12,478	50.00	60.98
NL63_R14	GAGTGCTGGRGAAGAAGCTATT	13,541	50.00	60.28
NL63_F15	GGTGTTCCACTTGTTACAACTGC	13,407	47.83	60.74
NL63_R15	CCTGTTAATGTTAGAACTNACGGC	14,493	45.83	60.22
NL63_F16	AGGCTTGGTAAYGAGTTGGCAC	14,358	50.00	61.32
NL63_R16	ACATCTAARGAACCAGTTGCTGAA	15,479	41.67	60.53
NL63_F17	GCATCAGGYTGTGGTGTTAGTGA	15,348	47.83	61.31
NL63_R17	TAATCRAYAGGCTCCATAACACCTTT	16,437	43.48	60.44
NL63_F18	TGCTGATATYGTTGTTGTAGATGAAGT	16,298	37.04	61.34
NL63_R18	ATTGTTCACCTGGTGGRGATTT	17,397	50.00	60.21
NL63_F19	GAAAGTGCTCATGTTTGTGG	17,259	50.00	61.35
NL63_R19	AACACAACTACTAGCRCGAAGG	18,353	50.00	61.10
NL63_F20	ACATGCRTTTCATACACCAGCA	18,233	45.45	61.31
NL63_R20	CAAAGTTGTGTCNGAAGCARTGAC	19,335	45.83	61.04
NL63_F21	GGAGTRTGATTTTCTTAACATRGAYATGGG	19,157	36.67	60.98
NL63_R21	GACAAAGACATAATAGTAGAATTACGCCAA	20,264	33.33	60.87
NL63_F22	GGACTTTGTTYTGCACNTCTGTT	20,137	43.48	60.92
NL63_R22	ATAAACAAGTTARACGRAGTGGTTG	21,203	52.17	61.13
NL63_F23	ATGTCACCACACNTAATGGCCG	21,048	50.00	61.45
NL63_R23	CAACTACCAGGCACTNCGACGGTTGA	22,171	52.38	61.24
NL63_F24	GAGTGNTTCACCAGGTGACTCTTC	22,069	50.00	61.41
NL63_R24	CAACATCAACAGTACCCAAACCAG	23,121	45.83	60.58
NL63_F25	AGCAATGCTTTTAGTTTGGCTAATGT	22,979	34.62	60.96
NL63_R25	ACACGAGATATGTTAACAAAAGT	24,062	31.13	60.82
NL63_F26	GGCTATGTNCTGCGTCAAYCTA	23,930	50.00	60.60
NL63_R26	ACCACCTAAAACGAYATARTGGTC	25,006	45.83	60.88
NL63_F27	TCGATTCATACATGTTGGCTATTATGC	24,855	37.04	60.63
NL63_R27	ATTAACAGARCGACCAACACGG	25,983	50.00	61.36
NL63_F28	GGTGTACTTCTTGTTGATGGCCA	25,879	47.83	61.31
NL63_R28	CTGTGGAAANCCYTTGGCATC	26,939	50.00	61.05
NL63_F29	TGATGGTGTTGTTTGGGTTGCT	26,415	50.00	60.98
NL63_R29	TGCGATCTTTAAGTACTCCAAC	27,479	34.48	61.37

* Nucleotide positions based on RefSeq hCoV genomes: NC_002645—229E, NC_006577—HKU1, NC_005831—NL63, and NC_006213—OC43.
