# Supplementary material for: Development of Primer Panels for Whole-Genome Amplification and Sequencing of Human Seasonal Coronaviruses: hCoV-OC43, hCoV-HKU1, hCoV-229E, and hCoV-NL63
Source: Viruses. 2024 Dec 25;17(1):13. doi: 10.3390/v17010013 (PMC11768711; doi:10.3390/v17010013)
Supplement: Supplementary file 1 [file viruses-17-00013-s001.zip › Supplementary_tables_S1a-S1d.pdf]

Table S1a. Distribution of hCoV-typed samples by epidemic seasons.

Epidemic season	hCoV-OC43 (n=130)	hCoV-NL63 (n=120)	hCoV-229E (n=50)	hCoV-HKU1 (n=30)
2017-2018	5/3.85	30/25.00	1/2.00	5/16.67
2018-2019	19/14.62	9/7.50	24/48.00	1/3.33
2019-2020	2/1.54	50/41.67	2/4.00	8/26.67
2020-2021	28/21.54	1/0.83	6/12.00	0/0.00
2021-2022	10/7.69	20/16.67	2/4.00	11/36.67
2022-2023	66/50.77	10/8.33	15/30.00	5/16.67

Table S1b. Distribution of hCoV-typed samples by patient gender.

Gender	hCoV-OC43 (n=130)	hCoV-NL63 (n=120)	hCoV-229E (n=50)	hCoV-HKU1 (n=30)
**Not NA**	**103/79.23**	**31/25.83**	**23/46.00**	**16/53.33**
Female	45/43.69	10/32.26	13/56.52	3/18.75
Male	58/56.31	21/67.74	10/43.48	13/81.25

Not NA —Samples with patient gender data.

Table S1c. Distribution of hCoV-typed samples by age groups.

Age group	hCoV-OC43 (n=130)	hCoV-NL63 (n=120)	hCoV-229E (n=50)	hCoV-HKU1 (n=30)
**Not NA**	**118/90.77**	**117/97.50**	**48/96.00**	**29/96.67**
0-2 years	69/58.47	85/72.65	26/54.17	13/44.83
3-6 years	24/20.34	14/11.97	11/22.92	6/20.69
7-17 years	21/17.80	1/0.85	3/6.25	3/10.34
18-64 years	4/3.39	15/12.82	6/12.50	7/24.14
≥65 years	0/0.00	2/1.71	2/4.17	0/0.00

Not NA — Samples with patient age data.

Table S1d. Shannon diversity index for all hCoV-typed samples by epidemic seasons.

Epidemic season	Total samples	Shannon index	Normalized Shannon index (1 — maximum possible variety)
2017-2018	41	0.83	0.60
2018-2019	53	1.10	0.80
2019-2020	62	0.66	0.48
2020-2021	35	0.58	0.42
2021-2022	43	1.19	0.86
2022-2023	96	0.94	0.68
