# Supplementary figures and images for: Development of Primer Panels for Whole-Genome Amplification and Sequencing of Human Seasonal Coronaviruses: hCoV-OC43, hCoV-HKU1, hCoV-229E, and hCoV-NL63
Source: Viruses. 2024 Dec 25;17(1):13. doi: 10.3390/v17010013 (PMC11768711; doi:10.3390/v17010013)

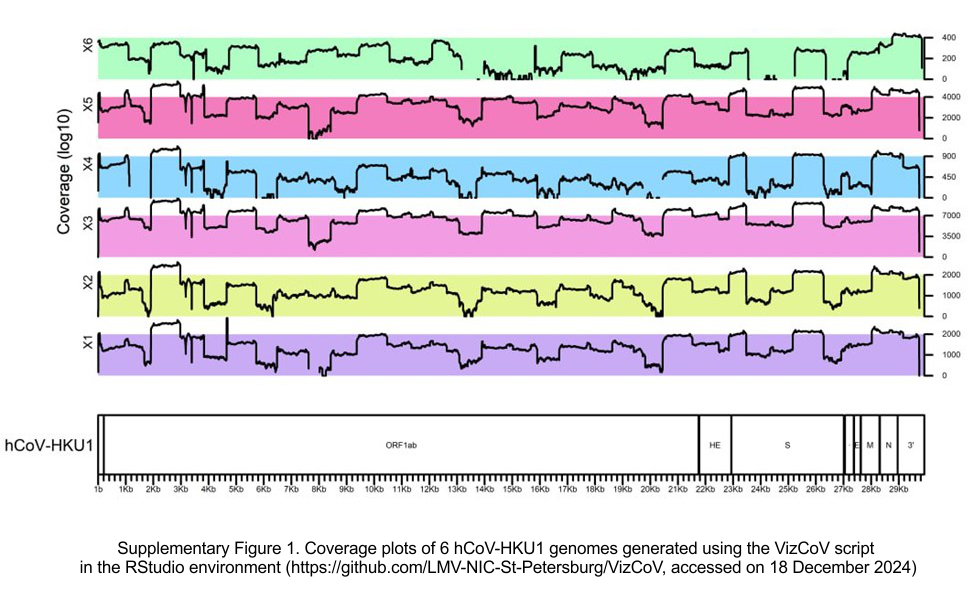

Supplement: Supplementary file 1 [file viruses-17-00013-s001.zip › Supplementary Fig. S1.png]

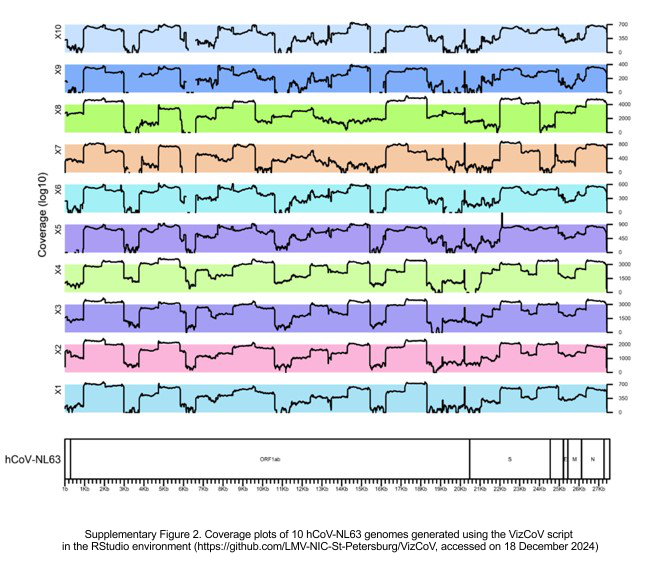

Supplement: Supplementary file 1 [file viruses-17-00013-s001.zip › Supplementary Fig. S2.png]

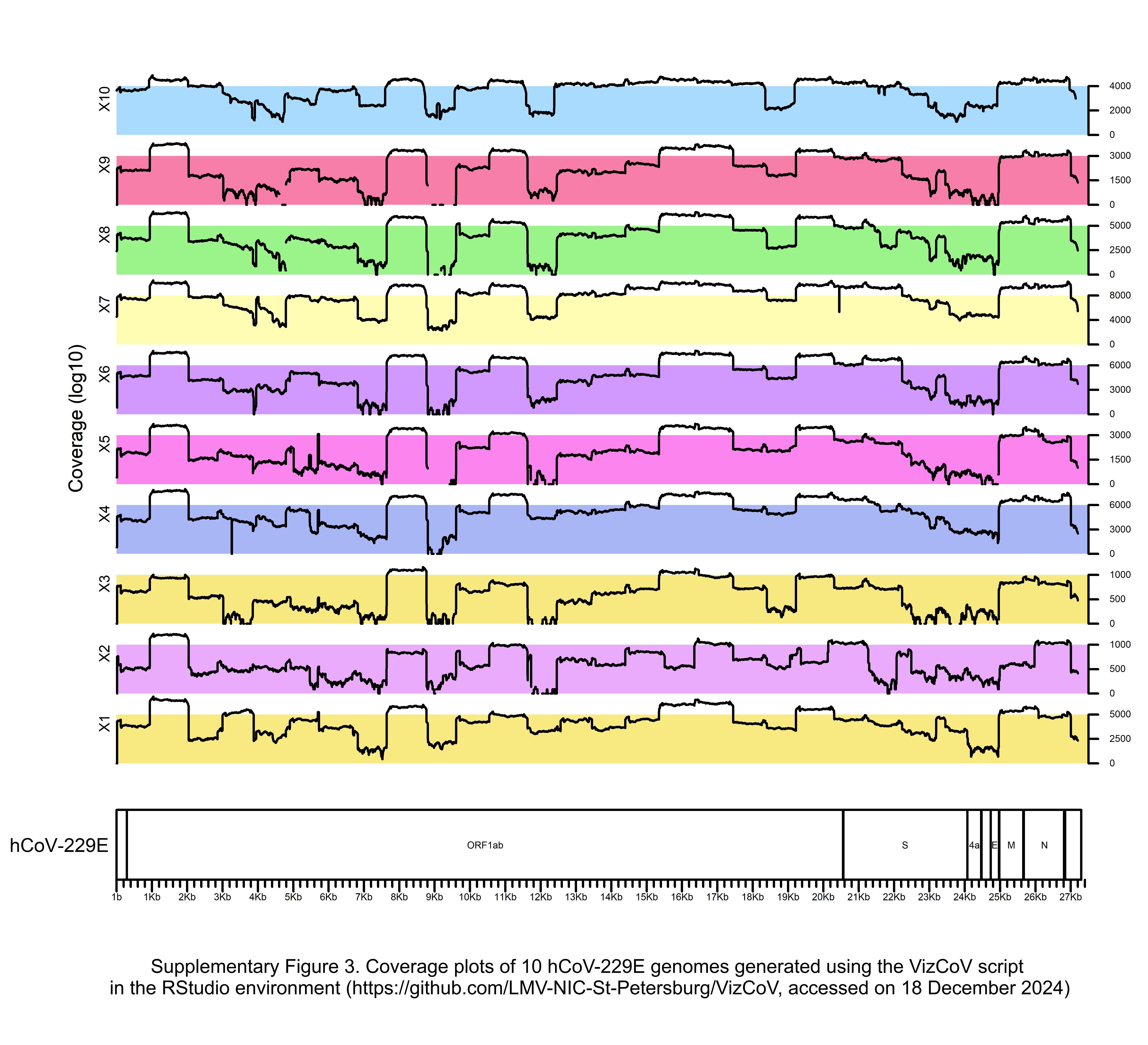

Supplement: Supplementary file 1 [file viruses-17-00013-s001.zip › Supplementary Fig. S3.png]

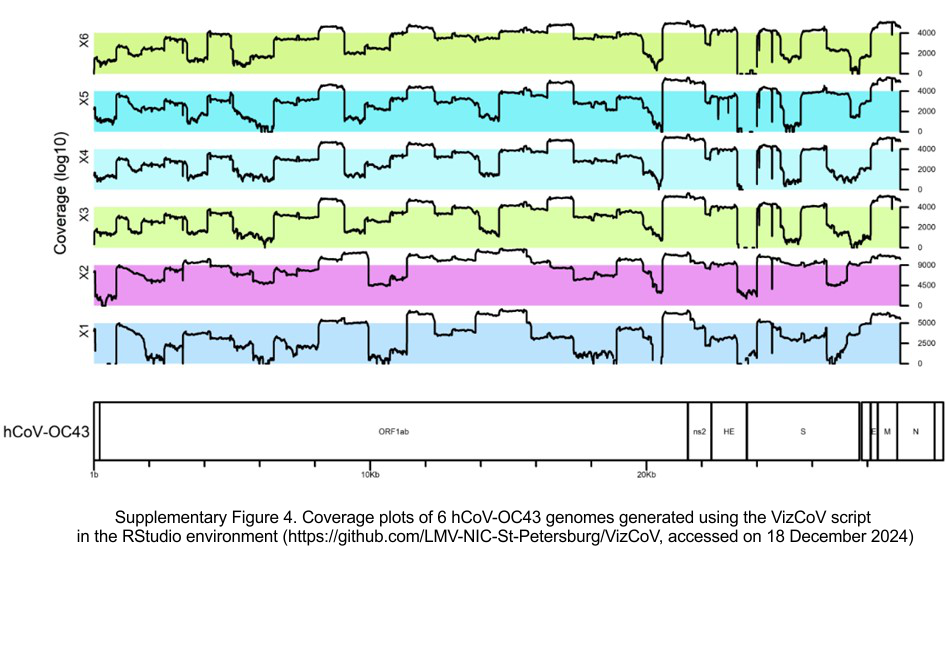

Supplement: Supplementary file 1 [file viruses-17-00013-s001.zip › Supplementary Fig. S4.png]

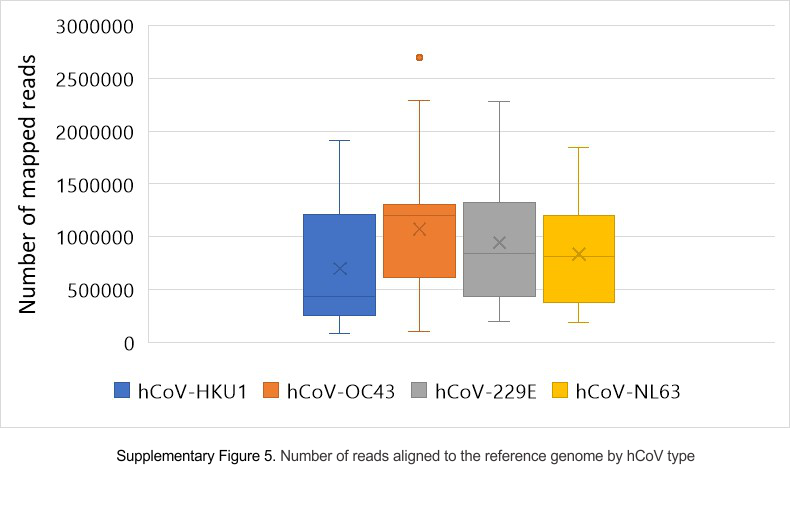

Supplement: Supplementary file 1 [file viruses-17-00013-s001.zip › Supplementary Fig. S5.png]
